# Study on Prestressed Concrete Beams Strengthened with External Unbonded CFRP Tendons

**DOI:** 10.3390/ma17184622

**Published:** 2024-09-20

**Authors:** Longlong Chen, Xuhong Qiang, Xu Jiang, Hao Dong, Wulong Chen

**Affiliations:** 1 College of Civil Engineering, Tongji University, Shanghai 200092, Chinaqiangxuhong@tongji.edu.cn (X.Q.);; 2 China Design Group Co., Ltd., Nanjing 210014, China

**Keywords:** prestressed concrete beam, CFRP tendon, reinforcement, finite element model, parametric study, practical design recommendation

## Abstract

This study builds a refined finite element (FE) model to research the flexural behavior of a reinforced beam with prestressed CFRP tendons. The precision of the FE model is validated through a comparison with the experimental outcomes. The numerical findings align well with the experimental outcomes, encompassing the failure mode, load-deflection curve, load-strain curves of concrete, steel reinforcements and CFRP tendons. The variances between predicted values and experimental results are within 10%. Leveraging the verified FE model, an extensive parametric study has been carried out to examine the effects of various parameters, including the CFRP tendon prestress, the CFRP tendon diameter, the deviator layout, the anchorage height and the prestressing strand prestress. Leveraging the findings from the parametric study, some refined design recommendations are proposed for practical reinforcement applications: Increasing the CFRP tendon prestress in practical reinforcement designs is recommended; CFRP tendons with larger diameters are recommended for use in practical reinforcement designs; Employing a linear CFRP tendon profile for reinforcement is not considered optimal in practical applications; The prestress loss in the prestressing strands of PC beams should be considered in practice.

## 1. Introduction

Prestressed concrete (PC) beams are extensively implemented in bridges [1,2,3,4,5] and industrial and civil buildings [6,7,8,9,10] due to their superior flexural behavior, crack resistance and high strength [11,12]. However, numerous PC beams in service suffer from various defects such as reduced stiffness, inadequate flexural resistance and pronounced deformation, due to the structural ageing, the concrete deterioration and the steel corrosion. The defects may result in serious structural damage [13]. Compared to reconstruction, strengthening the deteriorated beams represents a more economical solution, which can mitigate adverse impacts on society and the environment [14].

The most widely used reinforcement methods include the enlarging section method, near-surface mounted method, external bonding method and external unbonded prestressing method [13,14]. Compared to other techniques, the external unbonded reinforcement technology offers advantages such as secure connections, ease of construction and convenience in maintenance. This method involves arranging prestressed steel bars or strands along the sides or underside of the beam to narrow the crack width and improve flexural resistance [15,16]. Carbon fiber-reinforced polymer (CFRP) tendons are widely recognized as an ideal alternative to traditional prestressing steel bars for the prestressing reinforcement method, due to their advantageous properties such as reduced weight, exceptional strength, non-corrosive nature and excellent fatigue resistance [17,18,19].

Several studies have investigated the feasibility of employing CFRP tendons in the external unbonded reinforcement technique [20,21,22,23]. In a comprehensive experimental research work, Grace et al. [20] evaluated the static and fatigue performance of bridges reinforced with bonded internal CFRP tendons and unbonded external CFRP tendons. This experiment considered various factors, including the draping angle, deviator diameter, number of attached die-casts, etc. The prestress loss of the external unbonded CFRP tendons was found to be less than 7%, and the ductility of the reinforced bridge significantly improved at failure. Furthermore, they emphasized the importance of incorporating measures in deviator design to reduce the stress concentration on the CFRP tendons.

In an experimental research work conducted by El-Hacha et al. [21,22], twelve reinforced beams were tested to validate the effectiveness of the reinforcement method in improving the capacity and serviceability. The research delved into the impact of various parameters, including the span-depth ratio, the partial prestressing ratio, etc. The experimental outcomes revealed a significant enhancement, with the reinforced beams demonstrating an 83% higher ultimate load capacity and an improved overall performance. Fang et al. [23] conducted an investigation into the structural response of beams reinforced with prestressed CFRP tendons. Moreover, an analytical model with high accuracy was developed for calculating the behavior of the reinforced beams.

Therefore, the reinforcement method using an external unbonded CFRP tendon substantially improves the ultimate flexural resistance and ductility of concrete beams. There are some published numerical studies on concrete beams reinforced with prestressed CFRP tendons [24,25,26,27,28,29,30,31,32].

A finite element (FE) model was used by Cho et al. [24] to conduct a parametric study. The model precision was validated using test outcomes. The errors of the cracking, yielding and ultimate loads between the numerical and experimental results were within 9%. Liu et al. [25] explored the long-term performance of concrete beams reinforced with CFRP tendons via FE analysis, factoring in concrete creep, shrinkage and tendon relaxation. The model accuracy was confirmed by experimental outcomes, with an error of no more than 5%. Tran et al. [29] numerically investigated the bending response of dry key-jointed precast segmental concrete beams prestressed with external FRP tendons. The model validated by tests was used to conduct an intensive parametric analysis with a focus on the second-order effect.

Recently, an experimental study on PC beams reinforced with external unbonded CFRP tendons was conducted [33]. This paper extends this work by establishing an FE model, and the model was validated by experimental results. Utilizing the verified FE model, a comprehensive parametric study was executed to evaluate the effect of several key parameters. Based on the findings of this parametric study, some refined design suggestions were proposed to improve the practicality of the reinforcement method.

## 2. Experimental Work

**Figure 1:**
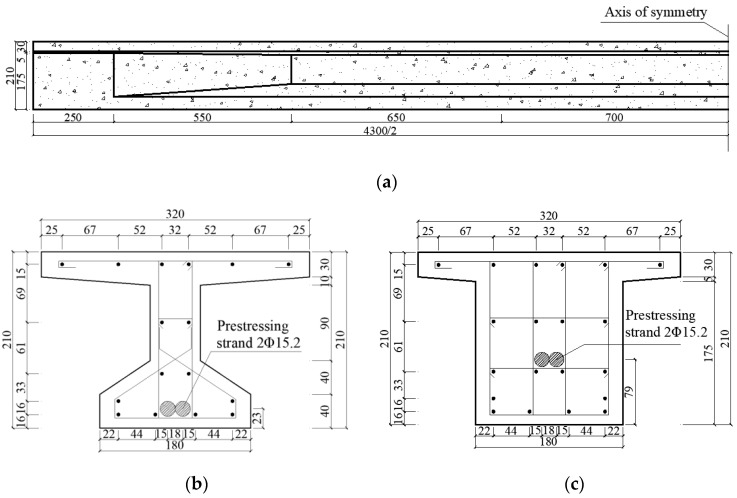
Size diagram of prestress concrete beam (unit: mm). (**a**) Front view; (**b**) Mid-span section; (**c**) Support section.

An experiment [33] was carried out to evaluate the correctness of the proposed FE model. In this study, the flexural behavior of PC beams reinforced with external unbonded CFRP tendons was investigated. The length and depth of the PC beam were 4300 mm and 210 mm, respectively, as illustrated in Figure 1a. The mid-span section of the beam was an I-shaped section, while the support section was a T-shaped section, as displayed in Figure 1b,c. The widths of the upper flange, web and lower flange of the mid-span section were 320 mm, 60 mm and 180 mm, respectively. The widths of the upper flange and web of the support section were 320 mm and 180 mm, respectively. The material mechanical properties of each component are shown in Table 1.

A total of four PC beams were fabricated, including one reference beam and three beams reinforced with CFRP tendons. The reinforcement schemes of the PC beams are detailed in Table 2. The PCB-U-0 was set as the reference beam, without any reinforcement measures. The prestress (*f_ci_*), the cross-sectional area (*A_f_*) and the initial tensile force (*T_f_*_0_) of the CFRP tendon were considered as the tested variables. The specimens PCB-S-1 and PCB-S-2 were reinforced with CFRP tendons with the same *A_f_* and a different *f_ci_* to study the effect of *f_ci_* on the flexural performance. The *A_f_* and *f_ci_* of PCB-S-3 were about two times those of the PCB-S-1, and the *T_f_*_0_ of PCB-S-3 was four times that of PCB-S-1. By comparing the flexural behavior of PCB-S-1 with that of PCB-S-3, the reinforcement effect of the *T_f_*_0_ could be explored.

**Figure 2:**
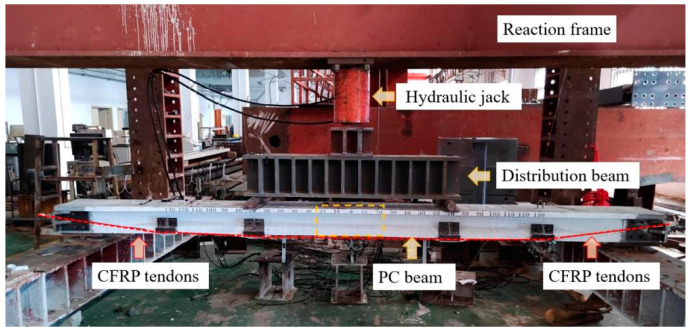
Test setup of the reinforced PC beam.

**Figure 3:**
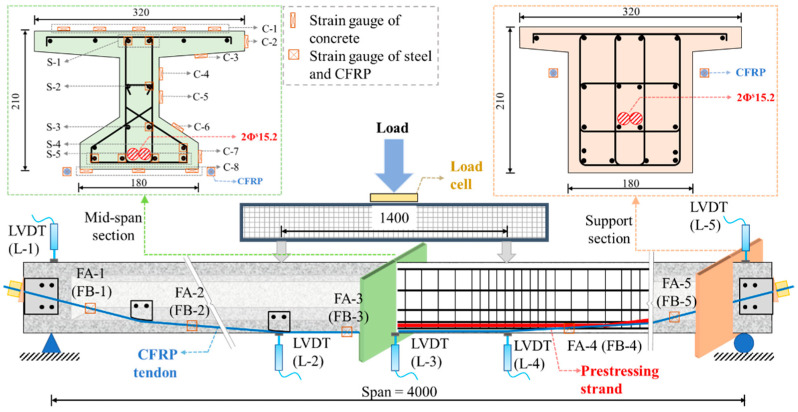
Schematic diagram of the reinforced beam (unit: mm).

A four-point bending test was carried out to examine the flexural performance of the PC beams. A 200-ton hydraulic jack was utilized to impose an external load, which was then conveyed via a 1.6 m distribution beam. A loading cell was employed for measuring the external load, and five linear variable displacement transducers (LVDTs) were used to monitor the deflection. Strain gauges were used to measure the strains of the concrete, steel reinforcement and CFRP tendon. The loading rate was approximately 0.2 kN/s, and the loading increment was 10 kN before cracking and 5 kN after cracking. The test setup is illustrated in Figure 2, and the schematic diagram for the reinforced PC beam is displayed in Figure 3. For further details, refer to [33].

## 3. Numerical Model

### 3.1. FE Model

**Figure 4:**
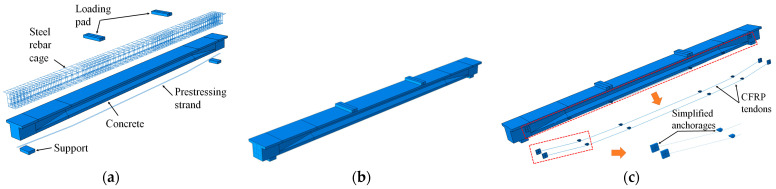
FE models of PC beams. (**a**) Explosive view of the reference beam; (**b**) Assembly view of the reference beam; (**c**) Assembly view of the reinforced beam.

In this research, a numerical investigation is conducted using the software Abaqus 2020 [34]. The exploded and assembly views of the FE models of both the reference and reinforced beams are shown in Figure 4. The FE model of the reference beam faithfully reflects the geometry of the PCB-U-0 beam described in Section 2, while the anchorage details of the reinforced beam models are simplified. The nomenclature of the FE models corresponds to that of the experimental specimens.

### 3.2. Element Types and Mesh

**Figure 5:**
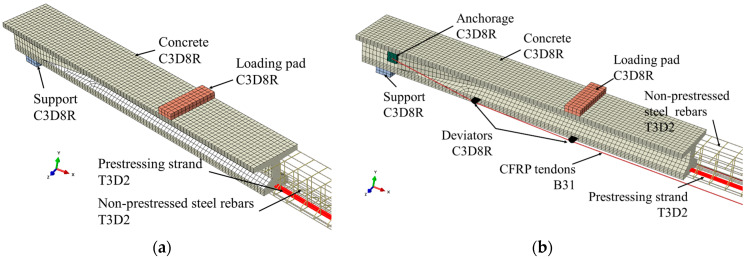
Element type and mesh of the FE models. (**a**) The reference beam; (**b**) The reinforced beam.

Figure 5 illustrates the types of elements utilized for each component. Concrete, loading pads, steel supports and simplified anchorages are modeled using an 8-node brick element with reduced integration (C3D8R). This element comprises eight nodes, each with three degrees of freedom [35]. Moreover, this element supports the analysis of plastic deformation [36], offering good accuracy at lower calculation costs during analysis [37].

Non-prestressed steel reinforcements and prestressing strands are simulated using a 2-node linear 3-dimensional truss element (T3D2). This element consists of two nodes, each with three degrees of freedom. CFRP tendons are modeled using 2-node linear beam elements (B31). This element comprises two nodes, each with six degrees of freedom [34]. Moreover, this element can better simulate the flexural response of the CFRP tendons at the bending point.

Each part of the FE model is built using an independent mesh. To strike a balance between result precision and analytical cost, a mesh sensitivity study based on the PCB-S-1 model is performed. The results are presented in Table 3. The numerical suffix of model nomenclature indicates the element mesh size of key components, including concrete, prestressing strands, steel reinforcements, supports and loading pads. For example, the PCB-S-1(20) indicates that the element mesh size of key components is 20 mm. The mesh size of other components, including the end anchorage and deviator, is appropriately adjusted according to their geometry and the mesh size of key components.

Table 3 demonstrates that the cracking load of PCB-C-1(25) matches the test result, with the ultimate load error kept within 5%. A mesh size smaller than 25 mm significantly increases the analysis time. Therefore, an optimal mesh size of 25 mm is recommended for key components. The mesh of the upper and lower flanges is locally refined in the vertical direction, and the mesh size is reduced to approximately 8 mm to ensure calculation accuracy. Additionally, the mesh size of the anchorage and deviator is set to 5 mm. It should be noted that the mesh size of these components is primarily based on their own dimensions and the dimensions of the contacting components, and their impact on the computational results is minimal.

### 3.3. Constraints, Boundary and Loading Conditions

**Figure 6:**
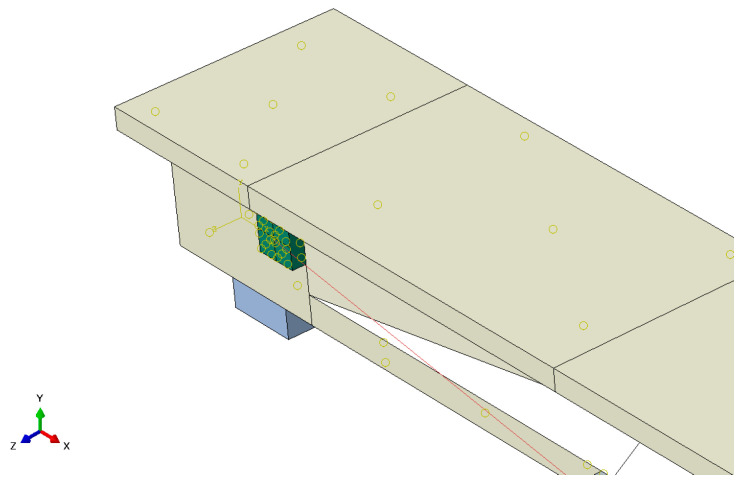
The connection type between the CFRP tendon and the end anchor.

**Figure 7:**
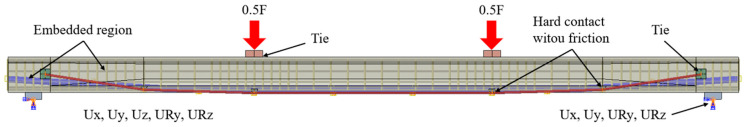
Constraints and boundary conditions of the FE model.

Prestressing strands and steel reinforcements are embedded within the concrete beam to ensure optimal bonding behavior between the embedded elements and the host elements [38]. Loading pads, supports, anchorages and deviators are connected to the concrete beam through “tie constraints”. The ends of the CFRP tendon are tied to the end anchorages, as shown in Figure 6, and the connection between the CFRP and deviator is modeled as a frictionless hard contact. The boundary condition of the FE model is configured as a simple-supported boundary, mirroring that of the test beam. The details of the constraints and boundary conditions are depicted in Figure 7.

Prestresses for both steel strands and CFRP tendons are achieved using the cooling method [39]. The gravity acceleration coefficient, representing the beam weight, is set as 9.8 m/s². Additionally, the external load is applied to loading pads.

Following the initial analysis step of the reference beam model, four static analysis steps are implemented to account for the self-weight of the concrete beam, the self-weight of the distribute beam, the prestress of strands and the external load. For the reinforced beam, an additional static analysis step, involving the prestress of the CFRP tendons, is introduced before applying the external load, resulting in a total of five static analysis steps.

### 3.4. Material Characteristics

#### 3.4.1. Steel

**Figure 8:**
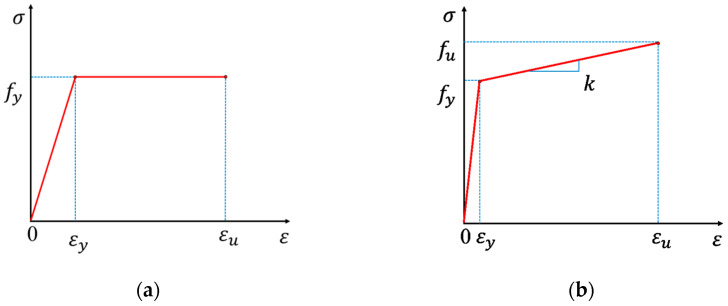
Stress-strain curve of steel constitutive models. (**a**) Ideal elastoplastic model; (**b**) Harding elastic-plastic model.

Figure 8 illustrates two common constitutive models of steel, including the ideal elastoplastic model and the hardening elastic-plastic model.

The ideal elastoplastic model is utilized for modeling the behavior of the non-prestressed steel reinforcement, as depicted in Figure 8a. Similarly, the loading pad, support, anchorage and deviator are also modeled using the ideal elastoplastic model. The relationship can be described as follows [40]:

(1) $$\sigma_{s}=\left\{\begin{array}{l} f_{y}\;\;\;\;\;\;\;\;\;\;\;\;\;\;\;\;\;\;\;\;\;\;\;\;\;\left(\varepsilon_{s}\geq \varepsilon_{y}\right)\\ E_{s}\varepsilon_{s}\;\;\;\;\;\;\;\;\;\;\;\;\;\;\;\left(-\varepsilon_{y}^{\prime }<\varepsilon_{s}<\varepsilon_{y}\right)\\ -f_{y}^{\prime }\;\;\;\;\;\;\;\;\;\;\;\;\;\;\;\;\;\;\;\left(\varepsilon_{s}\leq -\varepsilon_{y}^{\prime }\right) \end{array}\right.$$

where $\sigma_{s}$ is the stress of steel; $\varepsilon_{s}$ is the strain of steel; $E_{s}$ is the elastic modulus of steel; $f_{y},f_{y}^{\prime }$ are the yielding strength in tension and compression of the steel, respectively; and $\varepsilon_{y},\varepsilon_{y}^{\prime }$ are the yielding strain in tension and compression of the steel, respectively.

The hardening elastic-plastic model, as illustrated in Figure 8b, is employed for the prestressing strand. The relationship is as follows [40]:

(2) $$\sigma_{p}=\left\{\begin{array}{l} E_{p}\varepsilon_{p}\;\;\;\;\;\;\;\;\;\;\;\;\;\;\;\;\;\;\;\;\;\;\;\;\;\;\;\;\;\;\;\;\;\;\;\;\;\;\;\;\;\;\;\;\;\;\;\;\;\left(\varepsilon_{p}\leq \varepsilon_{py}\right)\\ f_{py}+k_{p}\left(\varepsilon_{p}-\varepsilon_{py}\right)\;\;\;\;\;\;\;\;\;\;\;\;\;\;\;\;\;\;\;\;\;\;\left(\varepsilon_{py}<\varepsilon_{p}\leq \varepsilon_{pu}\right) \end{array}\right.$$

(3) $$k_{p}=\frac{f_{pu}-f_{py}}{\varepsilon_{pu}-\varepsilon_{py}}$$

where $E_{p}$ is the elastic modulus of the strand; $f_{py}$ and $\varepsilon_{pu}$ are the yield and ultimate strength of the strand; $\varepsilon_{py}$ and $\varepsilon_{pu}$ are the yield and ultimate strain of the strand; and $k_{p}$ is the slope of the hardening line.

#### 3.4.2. Concrete

**Figure 9:**
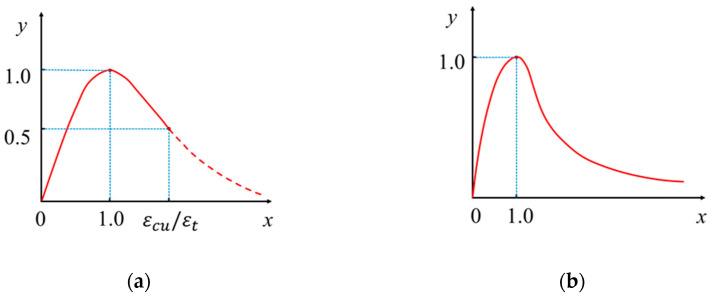
Stress-strain curve of the CDP model. (**a**) Compressive stress-strain relationship; (**b**) Tensile stress-strain relationship.

The behavior of concrete is simulated using the concrete damage plasticity (CDP) model. This model comprehensively accounts for the differences in the tensile and compressive behaviors of concrete by incorporating a material damage index. The elastic stiffness of the concrete is reduced based on the principle of strain equivalence, effectively modeling concrete stiffness degradation under loading [41]. The CDP model encompasses damage modes such as compressive failure and tensile cracking to accurately represent concrete behavior under various loading conditions. The relationship between concrete stress and strain is depicted in Figure 9.

The compressive stress-strain relationship of concrete is shown in Figure 9a. The relationship is as follows:

(4) $$y=\left\{\begin{array}{l} \alpha_{a}x+\left(3-2\alpha_{a}\right)x^{2}+\left(\alpha_{a}-2\right)x^{3}\;\;\;\;\;\;\;\;\;\;\;\;\;\;\;\;\;\;\;\;(x\leq 1)\\ \frac{x}{\alpha_{a}{\left(x-1\right)}^{2}+x}\;\;\;\;\;\;\;\;\;\;\;\;\;\;\;\;\;\;\;\;\;\;\;\;\;\;\;\;\;\;\;\;\;\;\;\;\;\;\;\;\;\;\;\;\;\;\;(x>1) \end{array}\right.$$

(5) $$y=\frac{\varepsilon }{\varepsilon_{cu}}$$

(6) $$x=\frac{\sigma }{f_{c}}$$

(7) $$\varepsilon_{cu}=\left(700-172\sqrt{f_{c}}\right)\times 10^{-6}$$

(8) $$\alpha_{a}=2.4-0.01f_{c}$$

(9) $$\alpha_{d}=0.132f_{c}^{0.785}-0.905$$

where $f_{c}$ is the concrete compressive strength; $\varepsilon_{cu}$ is the concrete strain corresponding to the compressive strength; and $\alpha_{a}$ and $\alpha_{d}$ are the parameters of the rising and falling stags of the compressive stress-strain curve of concrete, respectively.

The tensile stress-strain relationship of concrete can adopt the tensile model given in GB 50010-2010 [42], as shown in Figure 9b. The relationship is as follows:

(10) $$y=\left\{\begin{array}{l} 1.2x-0.2x^{6}\;\;\;\;\;\;\;\;\;\;\;\;\;\;\;\;\;\;\;\;\;\;\;\;\;\;\;\;(x\leq 1)\\ \frac{x}{\alpha_{t}{\left(x-1\right)}^{1.7}+x}\;\;\;\;\;\;\;\;\;\;\;\;\;\;\;\;\;\;\;\;(x>1) \end{array}\right.$$

(11) $$y=\frac{\varepsilon }{\varepsilon_{tu}}$$

(12) $$x=\frac{\sigma }{f_{t}}$$

(13) $$\varepsilon_{t}=65f_{t}^{0.054}\times 10^{-6}$$

(14) $$\alpha_{t}=0.312f_{t}^{2}$$

where $f_{t}$ is the concrete tensile strength; $\varepsilon_{tu}$ is the concrete strain corresponding to the tensile strength; and $\alpha_{t}$ is the parameters of the falling stag of the tensile stress-strain curve of concrete.

#### 3.4.3. CFRP

**Figure 10:**
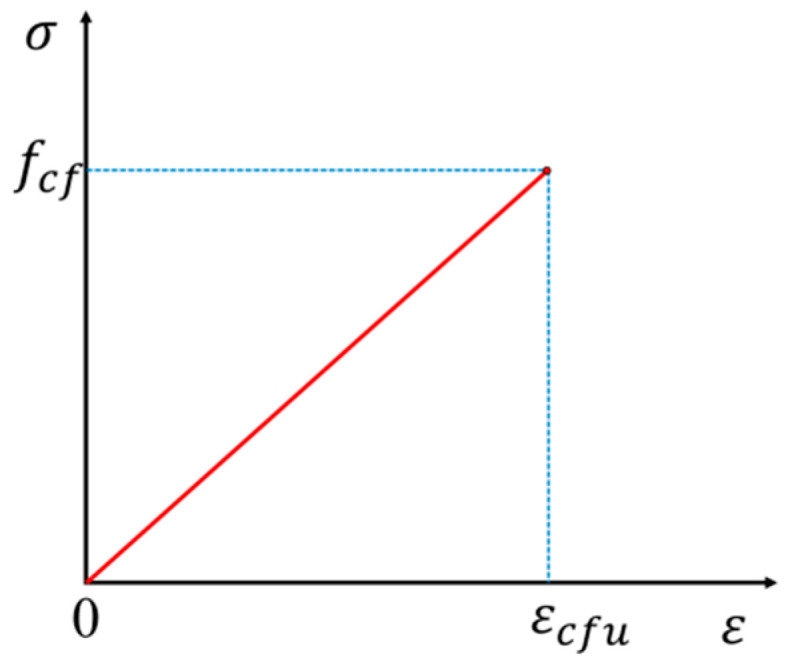
Stress-strain curve of CFRP.

CFRP tendons exhibit greater strength in the longitudinal direction compared to the radial direction [39]. The well-designed anchorages ensure that the CFRP tendons remain undamaged in the radial direction. Therefore, the stress-strain curve of the CFRP tendon in the longitudinal direction can be idealized as ideal elastic, as depicted in Figure 10.

The relationship of the curve can be described as follows:

(15) $$\sigma_{cf}=E_{cf}\varepsilon_{cf}$$

where $\sigma_{cf}$ is the CFRP tensile stress; $\varepsilon_{cf}$ is the CFRP strain; $E_{cf}$ is the CFRP elastic modulus; and $\varepsilon_{cfu}$ is the CFRP ultimate.

## 4. Validation of the Numerical Model

### 4.1. Primary Results

Table 4 presents the comparison between the numerical and experimental outcomes. It is observed from Table 4 that the error of the primary results does not exceed 10%.

### 4.2. Failure Mode

**Figure 11:**
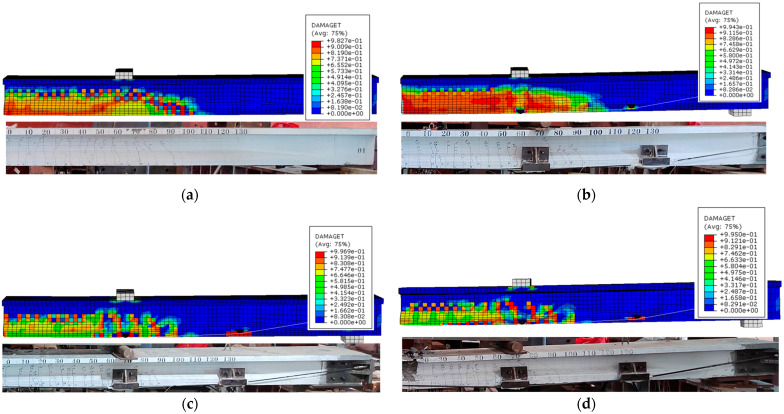
Comparison diagram of cracks. (**a**) PCB-U-0; (**b**) PCB-S-1; (**c**) PCB-S-2; (**d**) PCB-S-3.

**Figure 12:**
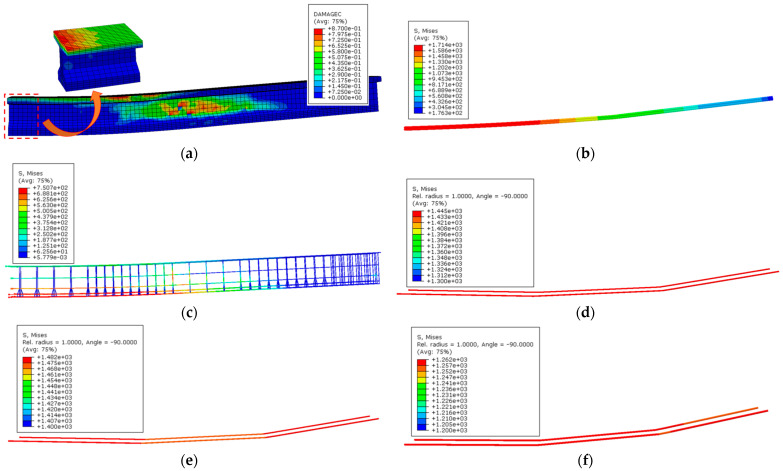
Comparison diagram of the FE models. (**a**) Concrete of the PCB-U-0; (**b**) Prestressing strands of PCB-U-0; (**c**) Non-prestressed steel reinforcements of PCB-U-0; (**d**) CFRP tendons of PCB-S-1; (**e**) CFRP tendons of PCB-S-2; (**f**) CFRP tendons of PCB-S-3.

The crack distribution of the FE models closely reflects that observed in the experimental beams, as depicted in Figure 11. The majority of cracks are localized to the constant moment region, while several cracks manifest in the bending shear zone near the loading points. At the mid-span section of the PCB-U-0 beam, concrete crushing is evident in the compressive zone, as illustrated in Figure 12a. The non-prestressed steel reinforcements reach their ultimate strength (see Figure 12b), and the prestressing strands yield (see Figure 12c). The failure mode of the FE model PCB-U-0 aligns with that observed in the experiment.

In the reinforced beams, the stress cloud diagrams of the non-prestressed steel reinforcements, concrete and prestressing strands are basically the same as that of the PCB-U-0 beam. The stress cloud diagrams of CFRP tendons at failure, depicted in Figure 12d–f, suggest that they do not attain their ultimate strength.

In conclusion, the cracks and failure modes observed in the FE models closely align with those observed in the test beams, confirming that the established model effectively represents the flexural behavior of the PC beam.

### 4.3. Load-Deflection Curves

**Figure 13:**
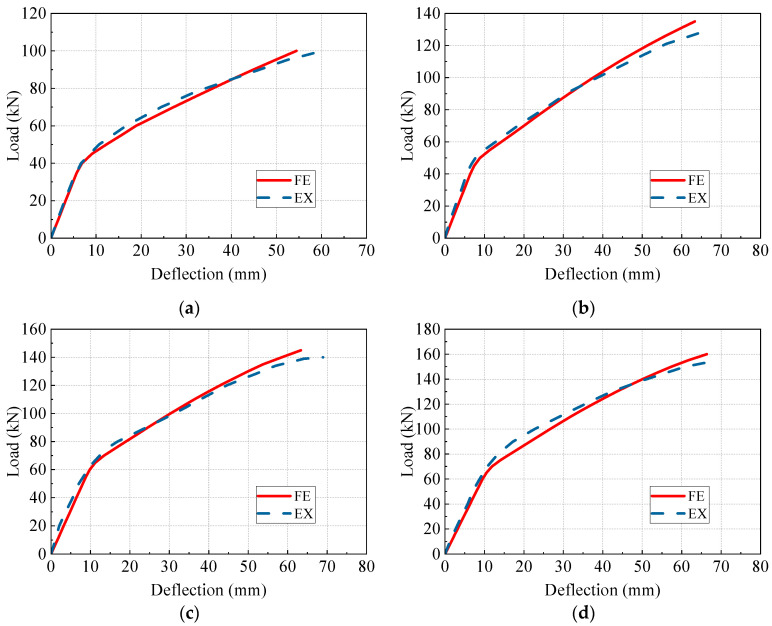
Comparison of load-deflection curves. (**a**) PCB-U-0; (**b**) PCB-S-1; (**c**) PCB-S-2; (**d**) PCB-S-3.

Figure 13 presents a comparative analysis of the load-deflection behaviors between the FE models and the experimental beams. In Figure 13, FE and EX denote the numerical and experimental curves, respectively. The numerical curves agree well with the experimental curves, indicating that the established FE model effectively simulates the flexural behavior of the PC beam and the reinforcement impact of the prestressed CFRP tendons. Furthermore, it is notable that the FE results exhibit slight differences from the experimental results, which may be attributed to variations in material properties or the boundary condition difference of the test beams and the FE models.

### 4.4. Load-Strain Curves

**Figure 14:**
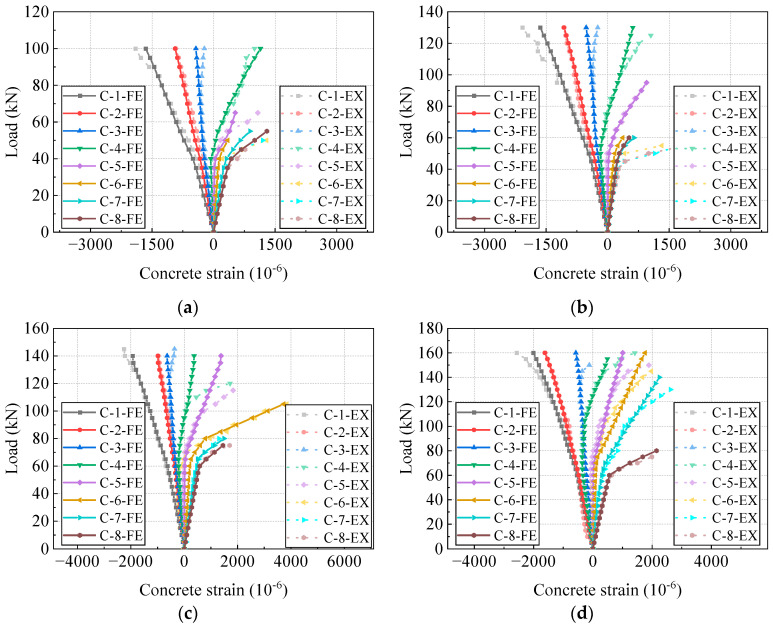
Comparison of load-strain curves of concrete. (**a**) PCB-U-0; (**b**) PCB-S-1; (**c**) PCB-S-2; (**d**) PCB-S-3.

**Figure 15:**
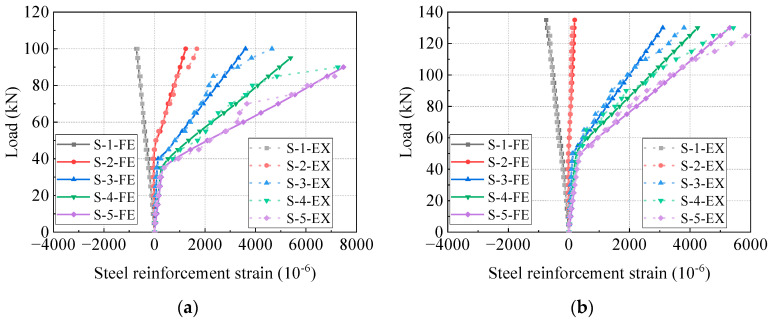

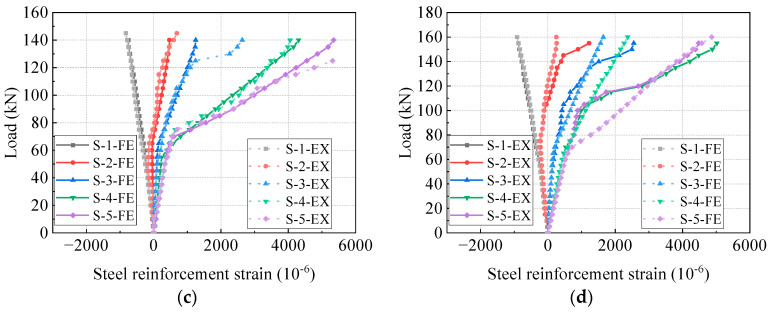
Comparison of load-strain curves of steel reinforcements. (**a**) PCB-U-0; (**b**) PCB-S-1; (**c**) PCB-S-2; (**d**) PCB-S-3.

**Figure 16:**
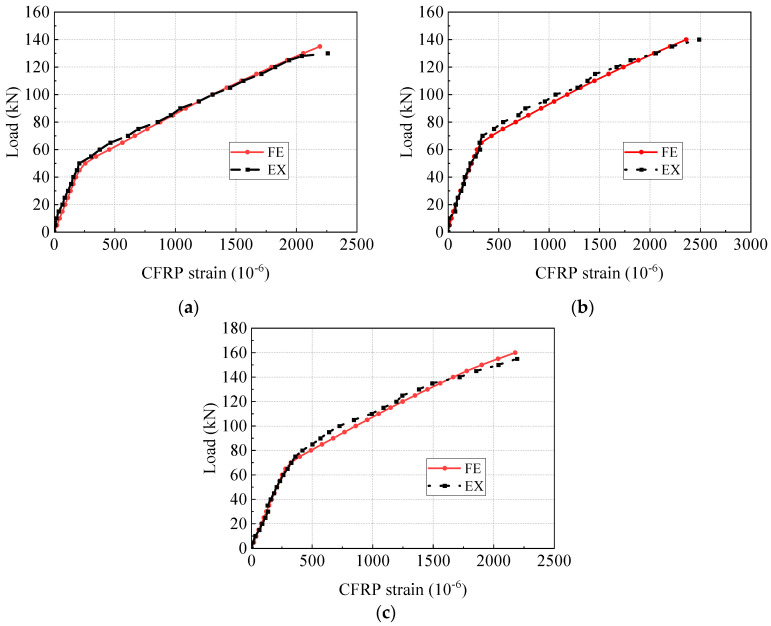
Comparison of load-strain curves of CFRP tendons. (**a**) PCB-S-1; (**b**) PCB-S-2; (**c**) PCB-S-3.

Figure 14 and Figure 15 compare the load-strain curves of concrete and steel reinforcements at the mid-span section between the FE models and the test beams. The location of strain gauges for concrete and steel reinforcements is depicted in Figure 3. The load-strain behaviors of concrete and steel reinforcements align closely. Figure 16 compares the load-strain curves of CFRP tendons between the FE models and the experimental beams. The numerical and experimental results show good agreement, indicating that the FE model can accurately forecast the strain development of the CFRP tendon.

## 5. Parametric Study

To further investigate the flexural performance of the reinforced beams, a parametric study is executed with the verified FE model. This analysis considers various parameters, including the diameter and prestress of the CFRP tendon, deviator layout, end anchorage position and prestress of the prestressing strands. Each parameter is studied individually while keeping other parameters constant. In this study, 31 FE models have been constructed, with the model parameters and corresponding calculation results detailed in Table 5.

### 5.1. Effect of CFRP Tendon Prestress

**Figure 17:**
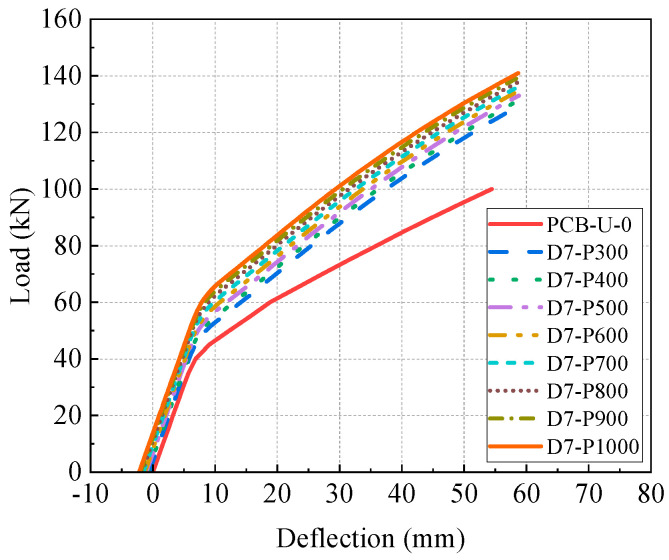
Load-deflection curves of beams with different CFRP prestresses.

Figure 17 illustrates the load-deflection curves of models with different prestresses of the CFRP tendon, considering the initial deflection after reinforcement. The load-deflection curves show two distinct stages: an elastic and a post-cracking stage. An obvious inflection point between two stages reflects the appearance of concrete cracking in the tensile zone.

**Figure 18:**
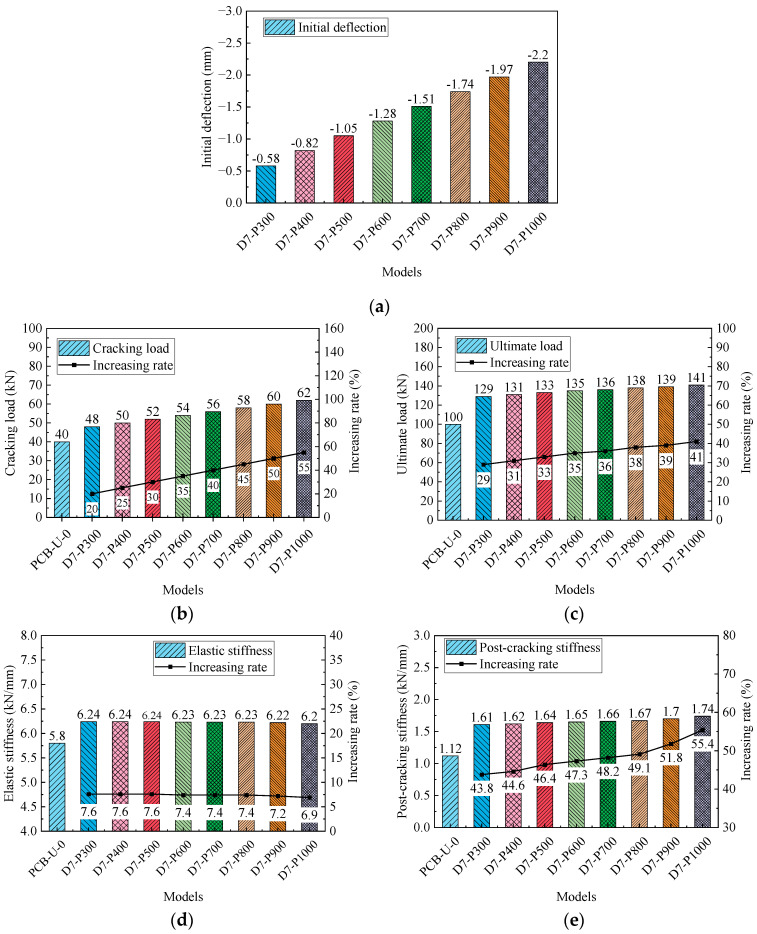
Primary results of beams with different CFRP prestresses. (**a**) Initial deflection; (**b**) Cracking load; (**c**) Ultimate load; (**d**) Elastic stiffness; (**e**) Post-cracking stiffness.

Figure 18 presents the primary results and their increasing rates of FE models. The results include the initial deflection, initial stiffness, post-cracking stiffness, cracking load and ultimate load. The initial deflection of reinforced models gradually increases with higher prestress of the CFRP tendon, resulting in a leftward shift of the load-deflection relationship in Figure 17. The post-cracking stiffness significantly improves with increasing CFRP tendon prestress, while elastic stiffness shows a slight decrease. Both the cracking load and ultimate load increase linearly with increasing prestress, and the increasing rate of the ultimate load is lower than that of the cracking load. The increasing rates of the cracking load and ultimate load for beam D7-P1000 are 55% and 41%, respectively. Compared to beam D7-P300, the prestress of beam D7-P1000 is increased by 233.3%, while the cracking load and the ultimate load are enhanced by 29.2% and 9.3%, respectively. This suggests that the influence of the prestressing of CFRP tendons on the ultimate load of the beam is relatively minor.

In summary, higher prestress levels of the CFRP tendon significantly improve the initial deflection, cracking load and ultimate load, with a negligible impact on elastic stiffness. Consequently, increasing the prestress of the CFRP tendon in practical reinforcement designs is recommended to enhance the performance of the reinforced beams.

### 5.2. Effect of CFRP Tendon Diameter

**Figure 19:**
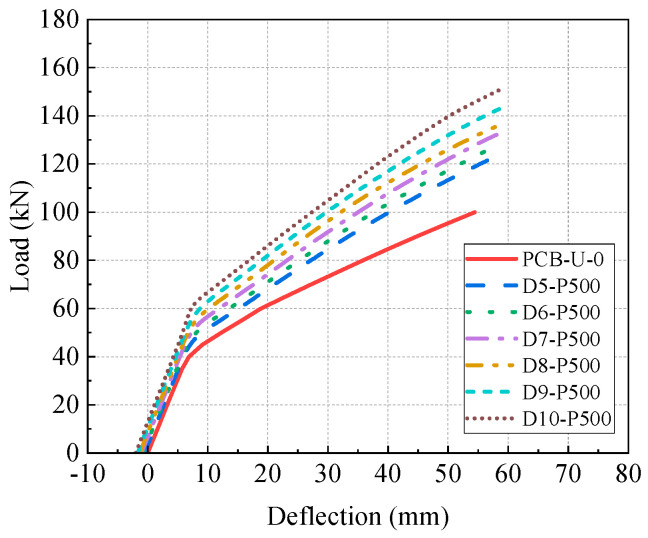
Load-deflection curve of beams with different CFRP diameters.

Figure 19 displays the relationship between the load and deflection for the beams with different diameters of CFRP tendons. The diameter of the CFRP tendon notably influences the flexural performance of the reinforced beams. Moreover, the ultimate deflection of the beam is not adversely affected by the CFRP tendon.

**Figure 20:**
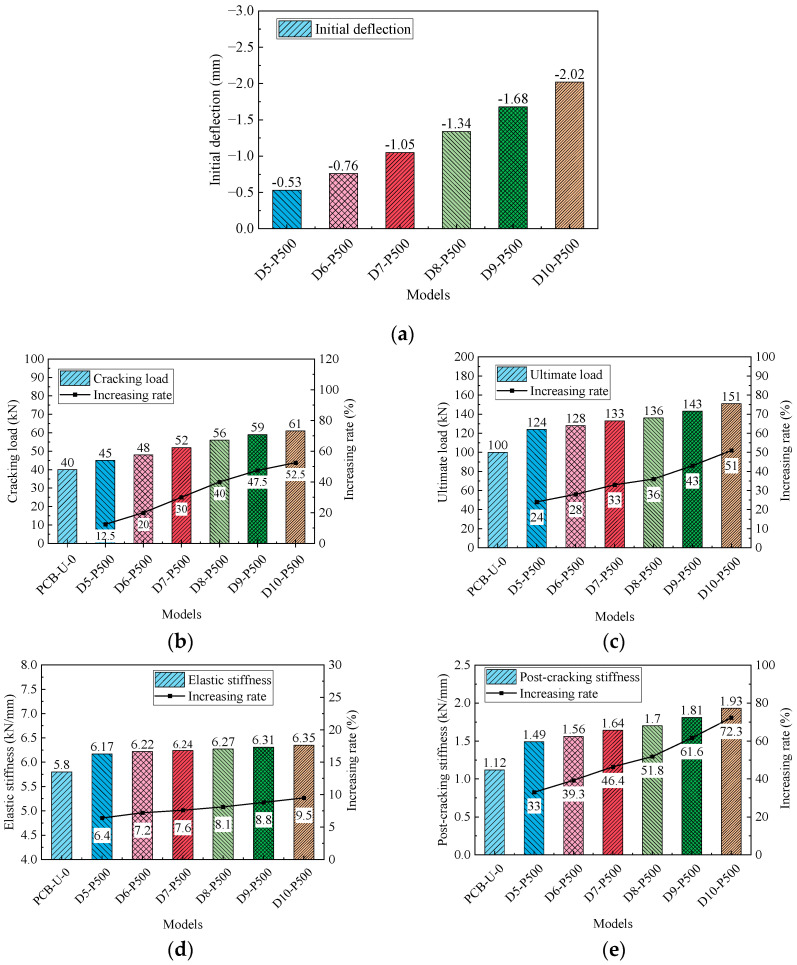
Primary results of beams with different CFRP diameters. (**a**) Initial deflection; (**b**) Cracking load; (**c**) Ultimate load; (**d**) Elastic stiffness; (**e**) Post-cracking stiffness.

Figure 20 presents the primary results of the reinforced beams and their increasing rates compared to beam PCB-U-0. The initial deflection significantly increases with the increment in the CFRP tendon diameter. The initial deflection of beam D10-P500 reaches 2.02 mm. Both the cracking load and ultimate load show significant growth with increasing CFRP tendon diameters. Specifically, the increasing rates of cracking and ultimate load for beam D10-P500 are 52.5% and 51%, respectively. Increasing the CFRP tendon diameter substantially enhances the post-cracking stiffness of the PC beam and slightly improves elastic stiffness. The post-cracking stiffness of beam D10-P500 is 72.3% higher than that of beam PCB-U-0, primarily due to the increased moment of inertia of the cross-section resulting from the larger CFRP tendon diameter.

Furthermore, the CFRP tendon tensile forces of beams D7-P1000 and D10-P500 are approximately equal. The increasing rate of the ultimate load of beam D10-P500 is bigger than that of beam D7-P1000, which suggests that the CFRP tendon diameter has a greater impact on the ultimate load than the prestress of the CFRP tendon. Compared to beam D5-P500, the cross-sectional area of the CFRP tendons in beam D10-P500 is increased by 400%, resulting in a 35.6% increase in the cracking load and a 21.8% increase in the ultimate load. This indicates that the cross-sectional area of the CFRP reinforcement has a significant impact on the cracking load of the beam.

In summary, increasing the diameter of CFRP tendons significantly improves the cracking load, ultimate load and post-cracking stiffness of the PC beams. Therefore, employing CFRP tendons with larger diameters in practical reinforcement designs is advantageous; however, it may lead to higher material and fabrication costs.

### 5.3. Effect of Deviator Layout and CFRP Tendon Profile

**Figure 21:**
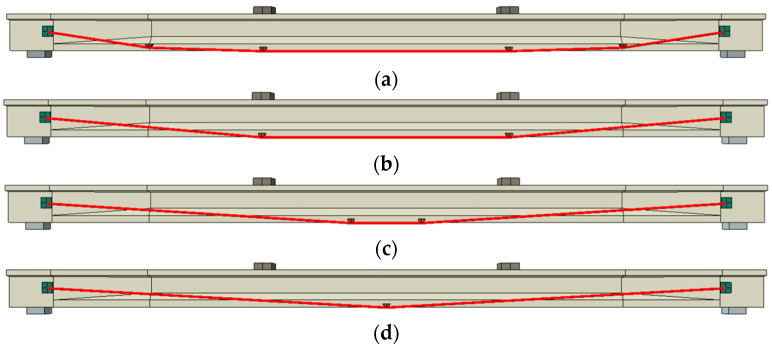
Schematic diagram of the deviator layout. (**a**) Z4-L1400; (**b**) Z2-1400; (**c**) Z2-L400; (**d**) 1-L0.

The profile of CFRP tendons is primarily adjusted by varying the deviator layout. The configurations of deviators and the profile of the CFRP tendon are presented in Figure 21. Beam Z2-L1400 has two deviators on one side of the CFRP tendon (see Figure 21a). The length of the horizontal segment of the CFRP tendon is 1400 mm, matching that of beam Z4-L1400. The primary difference among beams Z2-L1400 to Z2-L400 is the length of the horizontal segment of the CFRP tendon, as displayed in Figure 21b,c. In contrast, beam Z1-L0 includes only one deviator, and the CFRP tendon is divided into two inclined segments (see Figure 21d).

**Figure 22:**
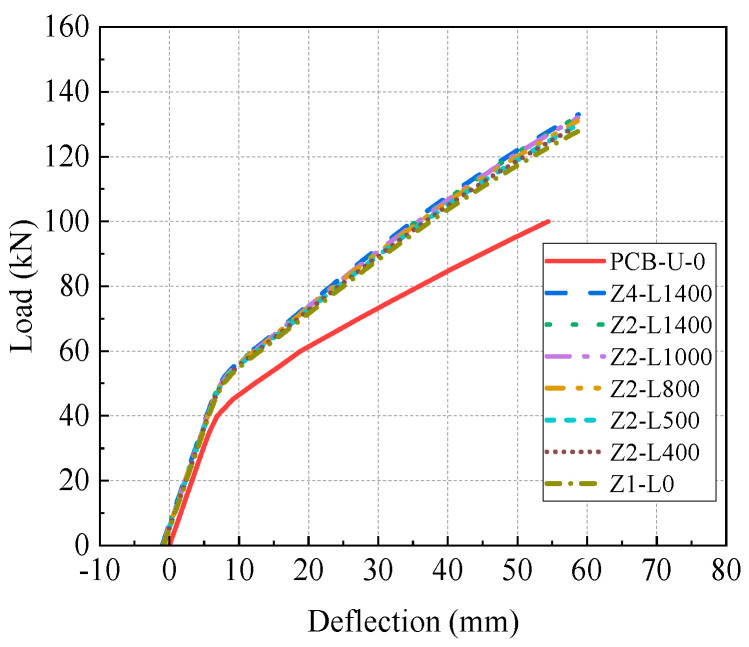
Load-deflection curve of beams with different deviator layouts.

**Figure 23:**
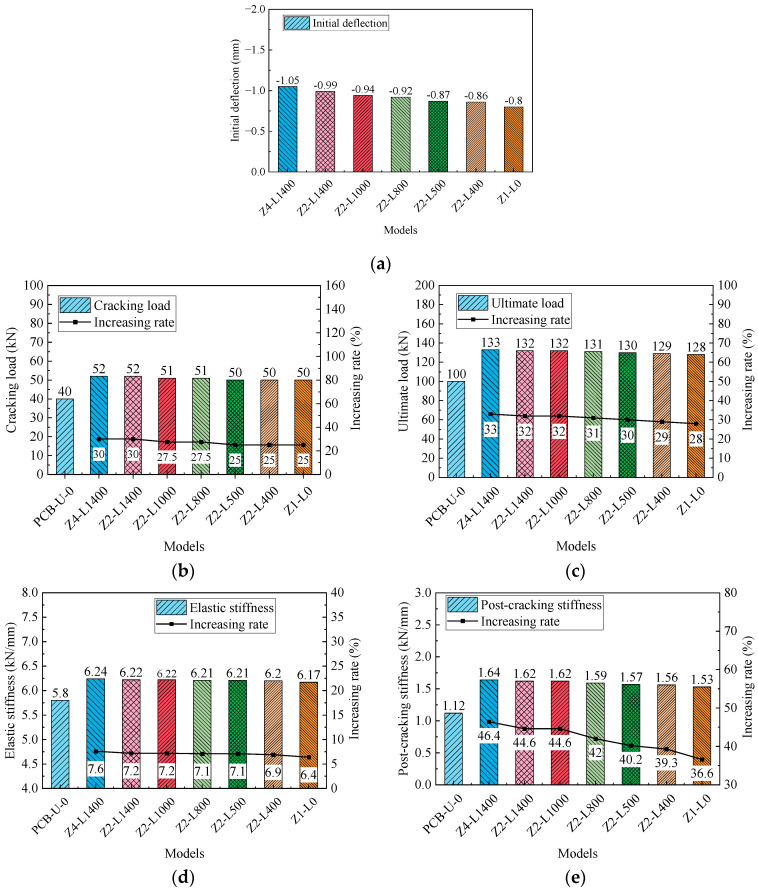
Primary results of beams with different deviator layouts. (**a**) Initial deflection; (**b**) Cracking load; (**c**) Ultimate load; (**d**) Elastic stiffness; (**e**) Post-cracking stiffness.

Figure 22 illustrates the relationship between the load and deflection for the beams reinforced with different deviator layouts. The slopes of the curves in the elastic stage are relatively consistent, whereas slight variations are noticeable in the post-cracking stage curves. Figure 23 presents the primary results and their increasing rates. The initial deflection, cracking load and ultimate load of the models decrease as the number of deviators is reduced and the horizontal segment length of the CFRP tendon shortens. More deviators offer increased resistance for the PC beam and enhance the overall deformation capability between the CFRP tendons and the PC beam. Similar to the load-deflection relationships, the variation in the elastic stiffness is negligible. Compared to beam Z2-L400, the length of the horizontal segment of the CFRP tendons in beam Z2-L1400 is increased by 250%; however, the cracking load and the ultimate load only increase by 3% and 2.3%, respectively. This suggests that the length of the horizontal segment has a minimal effect on both the cracking load and the ultimate load.

In summary, both reducing the number of deviators and shortening the CFRP horizontal segment length negatively affect the flexural behavior of reinforced beams, especially their post-cracking stiffness. Therefore, it is recommended to use more deviators and a longer horizontal segment of the CFRP tendon in practical reinforcement design, if feasible.

### 5.4. Effect of End Anchorage Height

**Figure 24:**
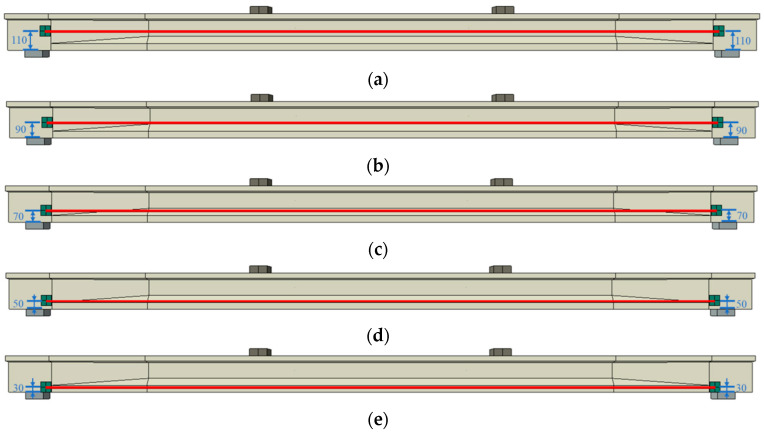
Schematic diagram of anchorage heights. (**a**) H110; (**b**) H90; (**c**) H70; (**d**) H50; (**e**) H30.

The linear CFRP tendon reinforcement system without a deviator differs from the system with a fold-line profile of CFRP tendons. Beams strengthened with linear CFRP tendons and different end anchorage heights are illustrated in Figure 24 to research the impact of anchorage heights on the flexural behavior of PC beams. The anchorage heights are marked in Figure 24, with a lower anchorage height positioned closer to the bottom of the PC beam.

**Figure 25:**
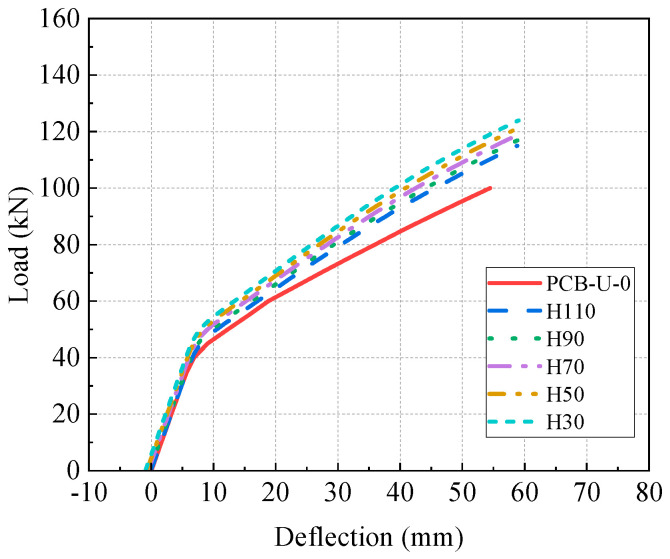
Load-deflection curve of beams with different anchorage heights.

**Figure 26:**
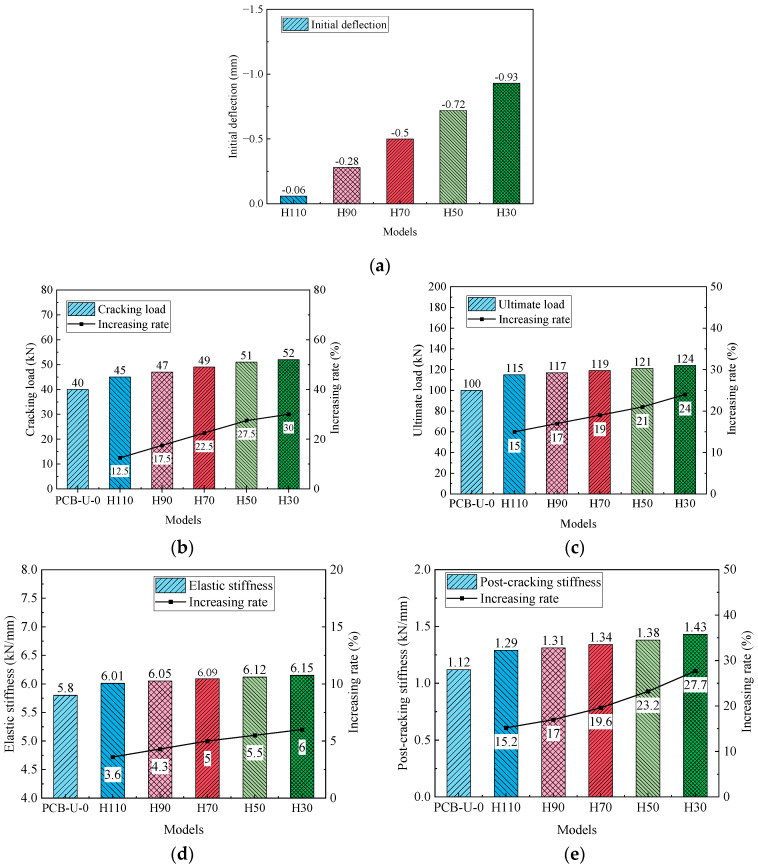
Primary results of beams with different anchorage heights. (**a**) Initial deflection; (**b**) Cracking load; (**c**) Ultimate load; (**d**) Elastic stiffness; (**e**) Post-cracking stiffness.

Figure 25 displays the relationship between the load and deflection for the beams with different anchorage heights. It is evident from Figure 25 that a lower anchorage height results in a higher ultimate load and greater post-cracking stiffness. Figure 26 presents the primary results and their increasing rates of the reinforced beams. Beams with lower CFRP tendon heights exhibit a greater initial deflection, cracking load and ultimate load. The CFRP tendon at the lower position can provide higher compressive stress to the concrete. Beam H30 shows an initial deflection of 0.93 mm, with increasing rates of the cracking load and ultimate load at 30% and 24%, respectively. Beam H30, reinforced with a linear CFRP tendon, shows a lower increase rate compared to beam D7-P500. This suggests that the reinforcement method using CFRP tendons with a fold-line profile is more effective. Furthermore, the post-cracking stiffness of the beam significantly increases as the anchorage height decreases, attributable to the lower anchorage position resulting in a larger moment of inertia. The post-cracking stiffness of beam H30 increases by 27.7% relative to beam PCB-U-0.

In short, the beams reinforced with lower anchorage positions exhibit superior flexural performance. Additionally, the method employing CFRP tendons with a fold-line profile offers a more effective reinforcement compared to the method using linear CFRP tendons. Thus, the reinforcement method utilizing linear CFRP tendons may not be the most effective approach.

### 5.5. Effect of the Strand Prestress

For deteriorated PC beams, prestress loss may occur in the embedded prestressing strands over their service life. Therefore, it is crucial to consider this prestress loss when evaluating the effectiveness of CFRP reinforcement. The strand prestresses for beams SP100 to SP900 are set at 100, 300, 500, 700 and 900 MPa, respectively. All beams are reinforced with CFRP tendons having a diameter of 7 mm and a prestress of 1000 MPa.

**Figure 27:**
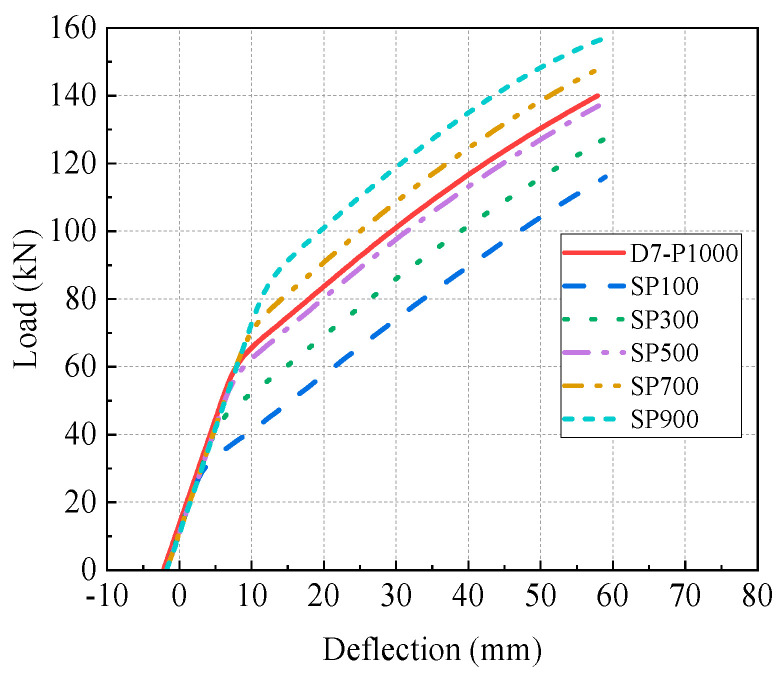
Load-deflection curve of beams with different strand prestresses.

**Figure 28:**
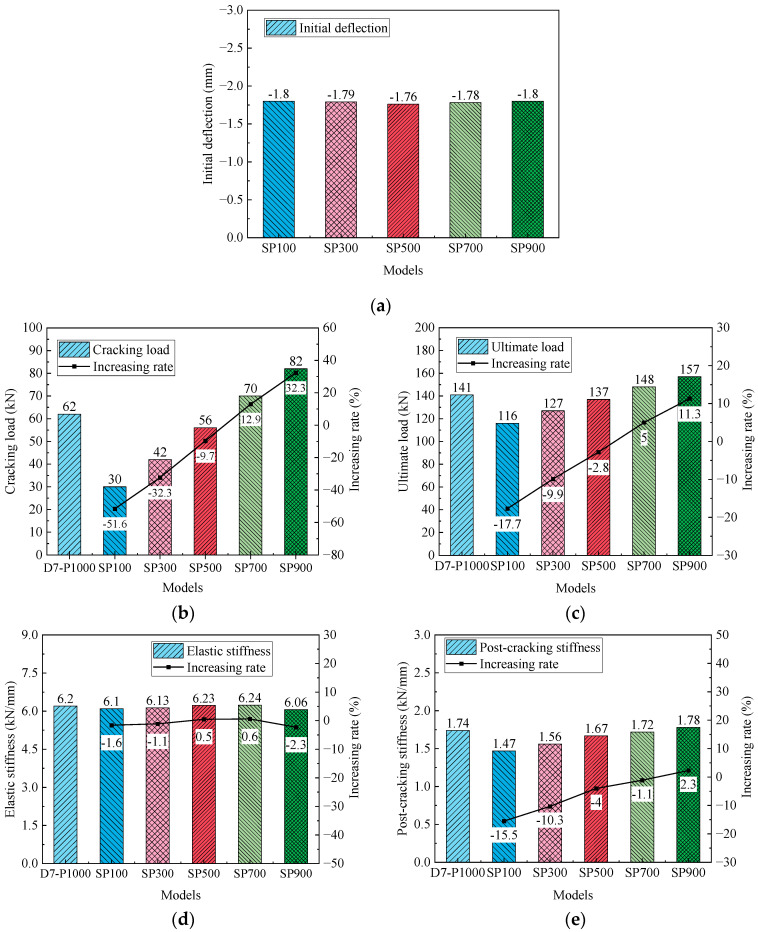
Primary results of beams with different strand prestresses. (**a**) Initial deflection; (**b**) Cracking load; (**c**) Ultimate load; (**d**) Elastic stiffness; (**e**) Post-cracking stiffness.

Figure 27 displays the relationship between the load and deflection for the reinforced beams. The primary results and their increasing rates of the reinforced beams are presented in Figure 28. The initial points of the load-deflection curves are generally consistent, suggesting that the effect of strand prestress on initial deflection can be negligible. However, the prestress of the prestressing strand notably influences the inflection point of the curves in Figure 27, suggesting that higher prestress leads to a greater cracking load in the reinforced beams. Additionally, the ultimate load increases linearly with an increment in the strand prestress. Specifically, the cracking and ultimate loads of beam SP100 are decreased by 51.6% and 17.7%, respectively, compared to those of beam D7-P1000. Figure 28d,e highlight the significant impact of prestress loss on cracking and ultimate loads, while its impact on elastic stiffness and initial deflection is minimal. In contrast to beam SP100, the prestress of the steel strands in beam SP900 is increased by 800%, yet the cracking load and the ultimate load are enhanced by 173.3% and 34.5%, respectively. This indicates that the prestress of the steel strands has a significant influence on the cracking load.

In summary, the prestress loss in the prestressing strand significantly impairs the effectiveness of the CFRP reinforcement, notably on the cracking load and ultimate load. Therefore, in practical reinforcement designs, the careful consideration of prestress loss is essential for the prestressing strand of PC beams.

## 6. Conclusions

This paper builds a refined FE model based on an experiment to evaluate the flexural performance of the PC beams reinforced with externally unbonded CFRP tendons. The accuracy of the FE model is verified using experimental outcomes. A subsequent parametric study is then conducted with the validated FE model, considering factors such as the CFRP tendon prestress, CFRP tendon diameter, deviator layout, anchorage height and prestress of the prestressing strand. This study leads to the following conclusions:

(1) The FE model accurately captures the flexural performance of the experimental beams, with the maximum error between the experimental results and the numerical outcomes not exceeding 10%. The failure modes, load-deflection curves and load-strain curves show good agreement between the FE models and experimental beams.

(2) Increasing prestress in the CFRP tendon significantly enhances the initial deflection, cracking load and ultimate load, with minimal effect on elastic stiffness. Compared to the reference beam, beam D7-P1000 showed increases of 55% in the cracking load and 41% in the ultimate load. When the prestress of CFRP tendons increased by 233.3%, the cracking load and ultimate load were enhanced by 29.2% and 9.3%, respectively. Therefore, increasing the prestress of the CFRP tendon in practical reinforcement designs is recommended.

(3) Increasing the CFRP tendon diameter significantly enhances the cracking load, ultimate load and post-cracking stiffness. Beam D10-P500 experienced a 52.5% increase in the cracking load and a 51% increase in the ultimate load, respectively. Furthermore, the CFRP tendon diameter has a more pronounced influence on the ultimate load than the CFRP tendon prestress level. When the cross-sectional area of the CFRP tendons is increased by 400%, the cracking load and the ultimate load are enhanced by 35.6% and 21.8%, respectively. Therefore, CFRP tendons with larger diameters are recommended for use in practical reinforcement designs.

(4) Reducing the number of deviators or shortening the CFRP horizontal segment length negatively impacts the flexural behavior of the reinforced beams, especially their post-cracking stiffness. When the length of the horizontal segment of the CFRP tendons is increased by 250%, the cracking load and the ultimate load only increase by 3% and 2.3%, respectively. In practical reinforcement designs, it is recommended to employ more deviators and a longer horizontal segment of the CFRP tendon, if possible.

(5) A beam reinforced with a lower anchorage position exhibits superior flexural behavior compared to one with a higher anchorage position. The flexural performance of a beam reinforced with a linear CFRP tendon is inferior to that of a beam strengthened with CFRP tendons featuring a fold-line profile. Therefore, employing a linear CFRP tendon profile for reinforcement is not considered optimal in practical applications.

(6) The prestress loss in the prestressing strand significantly affects the effectiveness of the external unbonded CFRP reinforcement method, particularly with respect to the cracking and ultimate loads. Compared to beam D7-P1000, beam SP100 shows a reduction of 51.6% in cracking load and of 17.7% in ultimate load. When the prestress of the steel strands is increased by 800%, the cracking load and the ultimate load are enhanced by 173.3% and 34.5%, respectively. Hence, in practical reinforcement designs, it is essential to account for the prestress loss in the prestressing strands of PC beams.

## Figures and Tables

**Table 1:** Mechanical properties of materials.

Components	Elastic Modulus (GPa)	Compressive Strength (MPa)	Tensile Strength (MPa)	Yield Strength (MPa)	Ultimate Strength (MPa)
Concrete	36.8	72	3.7	-	-
Steel rebar	196	-	-	724	752
Prestressing strand	201	-	-	-	1860
CFRP tendon	170	-	-	-	2300

**Table 2:** Test details of strengthened concrete beams.

Specimens	CFRP Tendons					Note
	Number	*D_f_* (mm)	*A_f_* (mm^2^)	*f_ci_* (MPa)	*T_f_*_0_ (kN)	
PCB-U-0	-	-	-	-	-	Reference beam
PCB-S-1	2	7	77.0	345	26.6	Reinforced beams
PCB-S-2	2	7	77.0	1150	88.5	
PCB-S-3	2	10	157.1	690	108.4	

Note: $D_{f}$ is the diameter of the CFRP tendon; $A_{f}$ is the cross-sectional area of the CFRP tendon; $f_{ci}$ is the prestress of the CFRP tendon.

**Table 3:** Results of the mesh sensitive study.

Models	Element Size (mm)	Element Number	Cracking Load (kN)			Ultimate Load (kN)			Analysis Time (s)
			*EX*	*FE*	Error (%)	*EX*	*FE*	Error (%)	
PCB-C-1(10)	10	194,992	50	49	−2	130	134	3.1	258,882
PCB-C-1(15)	15	99,083		49	−2		134	3.1	105,751
PCB-C-1(20)	20	70,299		50	0		135	3.8	34,344
PCB-C-1(25)	25	58,870		50	0		135	3.8	20,276
PCB-C-1(30)	30	51,870		51	2		137	5.4	16,545
PCB-C-1(35)	35	47,450		52	4		139	6.9	13,307
PCB-C-1(40)	40	43,910		54	8		140	7.7	11,903

Note: *EX* represents the experimental results; *FE* represents the numerical results.

**Table 4:** Comparison of the primary results.

Models	Elastic stiffness (kN/mm)			Cracking load (kN)			Ultimate load (kN)			Ultimate deflection (mm)		
	*EX*	*FE*	Error (%)	*EX*	*FE*	Error (%)	*EX*	*FE*	Error (%)	*EX*	*FE*	Error (%)
PCB-U-0	5.82	5.99	−2.8	40	40	0	100	100	0	54.44	60.17	−9.5
PCB-S-1	6.25	6.28	−0.5	50	50	0	135	130	3.8	63.34	67.17	−5.7
PCB-S-2	6.21	6.4	−3	70	70	0	145	140	3.6	63.2	68.98	−8.4
PCB-S-3	6.31	6.83	−7.6	75	80	−6.3	160	155	3.2	66.38	68.92	−3.7

Note: *EX* represents the experimental results; *FE* represents the numerical results.

**Table 5:** The parameters and results of the FE models.

Models	CFRP Tendon		Deviator Layout		Anchorage Position		Prestressing Strand	Numerical Results				
	Diameter (mm)	Prestress (mm)	Number	Middle CFRP Length	Height (mm)	Angle of CFRP (°)	Prestress (MPa)	Initial Deflection (mm)	Initial Stiffness (kN/mm)	Post-Cracking Stiffness (kN/mm)	Cracking Load (kN)	Ultimate Load (kN)
PCB-U-0	-	-	-	-	-	-	-	0	5.8	1.12	40	100
D7-P300	7	300	4	1400	116	5.11	580	−0.58	6.24	1.61	48	129
D7-P400		400						−0.82	6.24	1.62	50	131
D7-P500		500						−1.05	6.24	1.64	52	133
D7-P600		600						−1.28	6.23	1.65	54	135
D7-P700		700						−1.51	6.23	1.66	56	136
D7-P800		800						−1.74	6.23	1.67	58	138
D7-P900		900						−1.97	6.22	1.7	60	139
D7-P1000		1000						−2.2	6.2	1.74	62	141
D5-P500	5	500	4	1400	116	5.11	580	−0.53	6.17	1.49	45	124
D6-P500	6							−0.76	6.22	1.56	48	128
D7-P500	7							−1.05	6.24	1.64	52	133
D8-P500	8							−1.34	6.27	1.7	56	136
D9-P500	9							−1.68	6.31	1.81	59	143
D10-P500	10							−2.02	6.35	1.93	61	151
Z4-L1400	7	500	4	1400	116	5.11	580	−1.05	6.24	1.64	52	133
Z2-L1400			2	1400				−0.99	6.22	1.62	52	132
Z2-L1000			2	1000				−0.94	6.22	1.62	51	132
Z2-L800			2	800				−0.92	6.21	1.59	51	131
Z2-L500			2	500				−0.87	6.21	1.57	50	130
Z2-L400			2	400				−0.86	6.2	1.56	50	129
Z1-L0			1	0				−0.8	6.17	1.53	50	128
H110	7	500	0	2800	110	0	580	−0.06	6.01	1.29	45	115
H90					90			−0.28	6.05	1.31	47	117
H70					70			−0.5	6.09	1.34	49	119
H50					50			−0.72	6.12	1.38	51	121
H30					30			−0.93	6.15	1.43	52	124
SP100	7	1000	4	1400	116	5.11	100	−1.8	6.10	1.47	30	116
SP300							300	−1.79	6.13	1.56	42	127
SP500							500	−1.76	6.23	1.67	56	137
SP700							700	−1.78	6.24	1.72	70	148
SP900							900	−1.8	6.06	1.78	82	157

## Data Availability

Data are contained within the article.
